# An Optimization Study on Listening Experiments to Improve the Comparability of Annoyance Ratings of Noise Samples from Different Experimental Sample Sets

**DOI:** 10.3390/ijerph15030474

**Published:** 2018-03-08

**Authors:** Guoqing Di, Kuanguang Lu, Xiaofan Shi

**Affiliations:** College of Environmental and Resource Sciences, Zhejiang University, No. 866 Yuhangtang Road, Hangzhou 310058, China; 21514024@zju.edu.cn (K.L.); 21714034@zju.edu.cn (X.S.)

**Keywords:** noise annoyance, listening experiment, reference sound sample, calibration

## Abstract

Annoyance ratings obtained from listening experiments are widely used in studies on health effect of environmental noise. In listening experiments, participants usually give the annoyance rating of each noise sample according to its relative annoyance degree among all samples in the experimental sample set if there are no reference sound samples, which leads to poor comparability between experimental results obtained from different experimental sample sets. To solve this problem, this study proposed to add several pink noise samples with certain loudness levels into experimental sample sets as reference sound samples. On this basis, the standard curve between logarithmic mean annoyance and loudness level of pink noise was used to calibrate the experimental results and the calibration procedures were described in detail. Furthermore, as a case study, six different types of noise sample sets were selected to conduct listening experiments using this method to examine the applicability of it. Results showed that the differences in the annoyance ratings of each identical noise sample from different experimental sample sets were markedly decreased after calibration. The determination coefficient (*R*^2^) of linear fitting functions between psychoacoustic annoyance (PA) and mean annoyance (MA) of noise samples from different experimental sample sets increased obviously after calibration. The case study indicated that the method above is applicable to calibrating annoyance ratings obtained from different types of noise sample sets. After calibration, the comparability of annoyance ratings of noise samples from different experimental sample sets can be distinctly improved.

## 1. Introduction

Environmental noise is a growing health hazard worldwide. Around 100 million people are exposed to road traffic noise above 55 dB L_den_ (day-evening-night equivalent level) in the European Union [1]. In China, approximately 26% of monitoring points exceed the noise limits of corresponding environmental noise function zones at night [2]. Environmental noise can cause a series of health problems, such as sleep disturbance [3,4], learning impairment [5,6,7], hypertension ischemic heart disease [8,9,10], etc.

Annoyance is a widely used indicator to study the effect induced by different noise sources on well-being [11]. Harris’ research showed that the annoyance caused by road traffic noise influenced health-related quality of life [12]. Licitra surveyed the dose-effect relationship between the percentage of high annoying (%HA) and L_den_ of railway noise in Pisa, Italy [13]. Recently, researchers have paid more attention to the combined effect of different noise sources on annoyance [14,15,16].

Several alternative ways are available to evaluate noise annoyance. Zwicker [17] put forward the psychoacoustic annoyance (PA) model in 1999. Then Di [18] improved the PA model further, considering the tonality of noise. Using this model, relative annoyance degrees of different noises could be calculated directly by acoustical parameters through Equations (1)–(4):

(1) $${\mathrm{PA}=N}_{5}\left(1+\sqrt{w_{S}{}^{2}{+w}_{\mathrm{FR}}{}^{2}{+w}_{T}{}^{2}}\right)$$

(2) $$w_{S}=\{\begin{array}{ll} \left(S-1.75\right)\times 0.25\mathrm{lg}\left(N_{5}+10\right) & S>1.75\\ 0 & S\leq 1.75 \end{array}$$

(3) $$w_{\mathrm{FR}}=\frac{2.18}{N_{5}{}^{0.4}}\left(0.4F+0.6R\right)$$

(4) $$w_{T}=\frac{6.41}{N_{5}{}^{0.52}}\times T$$

where PA is psychoacoustic annoyance; N_5_ is the percentile loudness in sone; w_S_ describes the effect of sharpness S (acum), w_FR_ describes the influence of fluctuation strength F (vacil) and roughness R (asper), and w_T_ describes the effect of tonality T (tu).

Actually, environmental noise annoyance is influenced by both acoustical and non-acoustical factors [19]. Acoustical factors, such as environmental noise levels, etc., contribute only a part to the variance of environmental noise annoyance. PA is an objective quantity calculated by acoustical parameters, which ignores the influence of non-acoustical factors. Moreover, the value of annoyance calculated by PA model has no upper bound and can increase endlessly with the increase of acoustical parameters such as loudness, etc. Hence, field surveys and listening experiments are used more often by researchers to obtain noise annoyance. The annoyance ratings obtained in field surveys (a long-term response to environmental noise in context conditions) may be more valid than the ones in laboratory (a short-term response to recorded noise in a laboratory condition), considering the exposure time and context. However, field surveys are usually disturbed by background noise in researching the effect induced by certain noise source [20]. Hence listening experiments are usually used in the research where field surveys cannot be carried out or the research focusing on the effect of single noise source. In a listening experiment, a stimulus including several noise samples will be recorded in advance. Then the stimulus (experimental sample set) will be played to participants who will be asked to give the annoyance rating after listening to each noise sample. The average value of all ratings from different participants for each noise sample (i.e., mean annoyance, MA) will be calculated after listening experiments.

MA is widely used in research on environmental noise [21,22,23]. However, the comparability of MA values between different studies is poor. For instance, for two similar transformer noises at about 55 dB(A), participants tended to scale a higher rating (MA > 8) in the experiment conducted on the noise sample set ranging from 30 dB(A) to 57 dB(A) [24], while a much lower rating was obtained (MA < 4) in another experiment conducted on the noise sample set ranging from 50 dB(A) to 75 dB(A) [23]. This indicated that MA values obtained in listening experiments could only evaluate the relative annoyance degrees among noise samples in the same experimental sample set. To compare relative annoyance degrees of any other noise samples, even for those that have already been evaluated in different experimental sample sets, an additional listening experiment should be conducted. This poor comparability makes it difficult for researchers to use the experimental data in published studies to carry out further relevant research.

The poor comparability may be related to the lack of reference sound samples in different experimental sample sets. As there were no reference sound samples, participants evaluated the annoyance rating of each noise sample only according to its relative annoyance degree among all samples in each experimental sample set. To determine annoyance ratings of noise samples in each experimental sample set better, the relative magnitude estimation method, which provided a reference sound sample with known annoyance rating as an anchor for participants, was developed and used [25,26,27]. If the reference sound sample is identical, the comparability of annoyance ratings of noise samples from different experimental sample sets could be good. However, it is almost impossible to find a reference sound sample which is suitable for all listening experiments.

Nilsson has ever focused on improving the comparability of annoyance ratings from different studies [28]. He put forward the concept of the pink noise equivalent sound level (PNE_annoy_) which used the sound level of an equally annoying pink noise to represent the annoyance rating of one noise sample. The annoyance ratings of noise samples were all indicated by PNE_annoy_ so that those from different experimental sample sets could be compared directly. However, the annoyance magnitude of noise samples could not be showed directly when PNE_annoy_ was used as the indicator of annoyance rating. It would be better to transform the PNE_annoy_ into the traditional MA value further as the annoyance ratings of noise samples.

This study proposed an improved method which can amend the comparability of annoyance ratings of noise samples from different studies (experimental sample sets). Furthermore, as a case study, several different types of noise sample sets were selected to conducted listening experiments using this method to examine the applicability of it. 

## 2. Calibration Method

In the method proposed in this study, a standard curve and a reference curve are needed to calibrate the MA of noise samples from different experimental sample sets. The reference curve is used to find a pink noise that is equally annoying with the noise sample. Then, the MA of this equally annoying pink noise will be determined using the standard curve. It is also the MA of this noise sample after calibration. After such calibration, the MA of noise samples from different experimental sample sets will be transformed into the values in the scale of the standard curve.

### 2.1. Standard Curve

The standard curve is used to scale the annoyance ratings of noise samples from different experimental sample sets. In this study, a linear fitting curve between logarithmic mean annoyance ratings (MA) and loudness level (L_N_) of pink noise will be used as the standard curve which can be obtained by a listening experiment conducted on a pink noise sample set, and it can be presented as Equation (5):

(5) $$\log_{10}{(\mathrm{MA})=a+\mathrm{bL}}_{N}$$

### 2.2. Reference Curve

In this method, several pink noise samples need to be added into each experimental sample set as reference sound samples. After listening experiments, a linear fitting curve between L_N_ and logarithmic MA of the reference sound samples can be obtained. This is used as the reference curve of the corresponding experimental sample set, and it can be presented as Equation (6):

(6) $$\log_{10}{(\mathrm{MA})=a}_{i}{+b}_{i}L_{N}$$

### 2.3. Calibration Procedure

The two curves (i.e., the standard curve and reference curve) above are used to calibrate the MA of noise samples from different experimental sample sets. The MA_after_ (MA after calibration) can be calculated with MA_before_ (MA before calibration) by Equation (7):

(7) $$\log_{10}{(\mathrm{MA}}_{\mathrm{after}})=a+\frac{b}{b_{i}}{(\log}_{10}{(\mathrm{MA}}_{\mathrm{before}}{)-a}_{i})$$

It should be noted that the item of log_10_ (MA) makes no sense when MA is equal to 0 in Equations (5)–(7). Therefore, it is assumed that a value of 0.01 is assigned to MA when MA is equal to 0.

**Figure 1:**
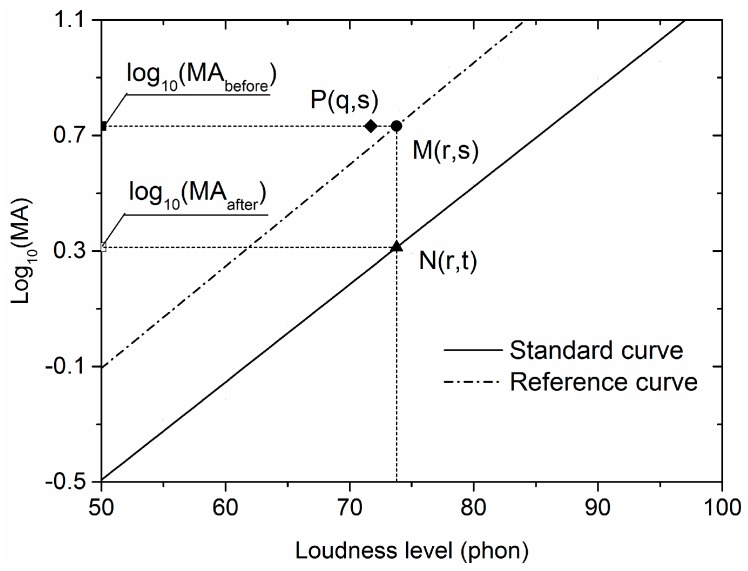
Illustration of calibration procedure of a demonstration sample (point P, shown as ◆ in the figure) for mean annoyance (MA).

As an illustration, Figure 1 shows the calibration procedure for the MA of a demonstration sample (point P, shown as ◆ in the figure). The coordinates of point P are (q, s). This means that the loudness level of the noise sample is q dB(A) and the logarithmic MA before calibration, i.e., log_10_(MA_before_), is s. In order to obtain the logarithmic MA after calibration, i.e., log_10_(MA_after_), two steps are needed.

**Step** **1** Find an equally annoying pink noise using the reference curve. Make a horizontal line through point P. It has a point of intersection (point M, showed as ● in the figure) with the reference curve (the dotted line in Figure 1). The coordinates of point M are (r, s). This means that the participants in this experiment think the noise sample is equally annoying (has an equal annoyance rating) with the pink noise at the loudness level of *r* dB(A).
**Step** **2** Determine the MA of this equally annoying pink noise in the scale of the standard curve. Make a vertical line through point M and get a point of intersection (point N, shown as ▲ in the figure) with the standard curve (the solid line in Figure 1). The coordinates of point N are (r, t). This means that in the scale of the standard curve, the logarithmic MA of this equally annoying pink noise is t, i.e., the logarithmic MA of the demonstration sample is s after calibration.

Following the two steps above, all MA of noise samples from different experimental sample sets can be transformed into the MA in the scale of the standard curve, which can improve the comparability of annoyance ratings of the noise samples from different experimental sample sets.

## 3. Case Study

### 3.1. Stimuli

The loudness range of noises used in listening experiments may vary. To assess whether our calibration method was effective in such research, six sets of noise samples (sample sets 1–6) with different loudness ranges were selected from a large database of recordings made with the Artificial Head Measurement System HMS IV.0 (HEAD acoustics GmbH, Herzogenrath, Germany). Each sample set had 12 five-second samples of noise. Half the sets (sample sets 1–3) were homogenous (transformer noise) and the others (sample sets 4–6) were heterogeneous (each set was composed of several kinds of noises).

The difference between annoyance ratings of an identical sample in different experimental sample sets is a good indicator to judge the comparability of experimental results; the smaller the difference, the better the comparability. Hence, several identical samples (samples A–E) were put into different noise sample sets (the identical samples were included in the 12 noise samples of each sample set). Table 1 shows the sources, loudness levels and energy distribution in different frequency ranges of the six identical noise samples. The energy distribution was calculated by Equation (8) in low-frequency range (20–200 Hz), middle-frequency range (200 Hz–2 kHz) and high-frequency range (2–20 kHz) [29]

(8) $$\eta_{k}=\frac{E_{k}}{E}=\frac{p_{k}{}^{2}}{p^{2}}{=10}^{0.1\left(L_{k}-L\right)}$$

where η_k_ is the sound energy proportion of low-, mid- or high-frequency range in the total sound energy; E_k_, p_k_, and L_k_ are the sound energy, sound pressure and sound pressure level of the corresponding frequency range, respectively; and E, p and L are total sound energy, total sound pressure, and total sound pressure level of noise sample, respectively.

As presented in Table 1, transformer noise and boiler noise are low-frequency noises, heat pump noise is mid-frequency noise, and the noise recorded in a workshop is high-frequency noise due to their dominant sound energy at the corresponding frequency ranges [30].

Additionally, seven pink noise samples were added into each sample set (sample sets 1–6) as reference sound samples. In each sample set, the range of loudness level of the added pink noise samples was a little wider than that of the 12 noise samples. The interval of loudness levels of two adjacent reference sound samples was equal. Considering that auditory discriminating thresholds of intensity were about 0.4 dB [31], the minimal interval of two adjacent reference sound samples was set to 0.5 phon. Thus, when the loudness levels of noise samples were identical, or the range of these loudness levels was smaller, the calibration method could also work well. Table 2 gives a detailed description of sample sets 1–6.

Another sample set (sample set 7) was composed of nine pink noise samples whose L_N_ ranged from 55 phon to 95 phon (A-weighted equivalent sound pressure level ranging from 38 dB(A) to 78 dB(A)). The interval of loudness levels between two adjacent pink noise samples was 5 phon. This sample set was used to establish the standard curve in this study. The pink noise samples used above were all generated automatically by ArtemiS 10.00 analysis software (HEAD acoustics GmbH, Herzogenrath, Germany).

In each sample set, all the noise samples were arranged randomly, and an interval of five s was inserted into every two noise samples, forming an evaluation sequence of noise samples. Three evaluation sequences with different orders were grouped together to be an experiment stimulus. Thus, seven sets of experiment stimuli were finally formed.

### 3.2. Apparatus and Setting

The binaural audio playback system consists of a digital equalizer (Head Acoustics PEQ V, HEAD acoustics GmbH, Herzogenrath, Germany), a distribution amplifier (Head Acoustics HDA IV. 1, HEAD acoustics GmbH, Herzogenrath, Germany) and four headphones (Sennheiser HD 600, Sennheiser electronic GmbH & Co. KG, Wedmark, Germany), which had already been calibrated at the calibration laboratory of Head Acoustics GmbH. All experiments were conducted in a soundproof room (3 m × 2 m × 3 m), where background noise was lower than 25 dB(A).

### 3.3. Procedure of Listening Experiments

The listening experiments were conducted separately for seven sample sets. In each experiment, 60 college students (22 males, 38 females, mean age of 24 years) with normal hearing condition were recruited randomly as participants. Due to the number of headphones in the binaural audio playback system, at most four participants could receive noise exposure at the same time. Before the experiment, participants were required to sit calmly on a chair, put on the headphones, and be ready for noise exposure. Then, the corresponding experiment stimulus was played back after previewing several pink noise samples. An 11-point numerical scale with continuous labels equally spaced from 0 (“not annoying at all”) to 10 (“extremely annoying”) was used for the annoyance evaluation of each noise sample in the interval. Since all participants in this study were Chinese college students with good English competence, the evaluation sheet was printed in both English and Chinese.

### 3.4. Statistical Analysis

Misjudgment is inevitable when the participants make a decision on the evaluation scores. Thus, it is necessary to examine the validity of the data. In this study, each participant was supposed to give three evaluation scores for each noise sample. The examination rule was that if the difference between any two of these three evaluation scores was within two, this result was accepted; otherwise, the three evaluation scores would be deleted.

According to the valid evaluation scores, mean annoyance (MA), as an indicator of annoyance response, was calculated by Equation (9) for each sound sample:

(9) $$\mathrm{MA}=\sum \left(n_{j}\times j\right)/\sum n_{j},$$

where j is a certain annoyance rating (0–10) in the numerical scale; and n_j_ is the total times of choosing j-th annoyance rating.

Psychoacoustic annoyance (PA) can well estimate the relative annoyance ratings of noise samples [17,18], so the relative magnitude of PA between noise samples is well consistent with the relative magnitude of MA obtained from listening experiments (i.e., the consistency of PA and MA is good). For this reason, PA of all noise samples was calculated by Equations (1)–(4) in this study. Then linear fit was performed between PA and MA for both individual sets (sample sets 1–6) and mixed sets (a set including the experimental results of several individual sets, e.g., a set including the experimental results of sample sets 1–3 was called mixed sets 1–3 for simplicity) before and after calibration. The determination coefficient (*R*^2^) was considered as a judgment index for the comparability of MA of noise samples. After calibration, if *R*^2^ increases, it means that the comparability of MA is improved. Contrarily, the comparability is reduced.

## 4. Results and Discussion

### 4.1. Linear Fitting Functions between L_N_ and Logarithmic MA

In Table 3, the linear fitting functions between L_N_ and logarithmic MA of noise samples in each sample set had excellent determination coefficients (*R*^2^: 0.875–0.982), which indicated a great correlation between L_N_ and MA of noise samples. Nilsson’s research also showed that L_N_ had the best correlation with annoyance of noise samples in all single acoustical factors [28]. Actually, the psychoacoustic annoyance (PA) model [17,18] also showed that loudness was the most important acoustical factor influencing the value of PA (see Equations (1)–(4)). Therefore, to calibrate the annoyance ratings of noise samples from different experimental sample sets, L_N_ was selected to establish a calibration method basing on the relationship between MA and L_N_ in this study, of course, basing on the relationship between another acoustical factor (e.g., L_A_) and MA, the calibration procedures described in chapter 2 could also be used to improve the comparability of MA obtained from different experimental sample sets. However, the calibration effectiveness could decrease. 

Theoretically, the linear fitting functions between L_N_ and logarithmic MA of pink noise in different sample sets with different ranges of L_N_ should be similar. However, as shown in Table 3, the linear fitting functions between L_N_ and logarithmic MA of pink noise in sample sets 1–7 (i.e., the standard curve and reference curves) were all different in this study. The main reason was that the participants were used to giving the annoyance rating of each noise sample according to its relative annoyance degree among all samples in the experimental sample set, which led to the poor comparability of MA of noise samples from different sample sets. In fact, it was also the reason why this study proposed a method to calibrate MA obtained from different experimental sample sets.

**Figure 2:**
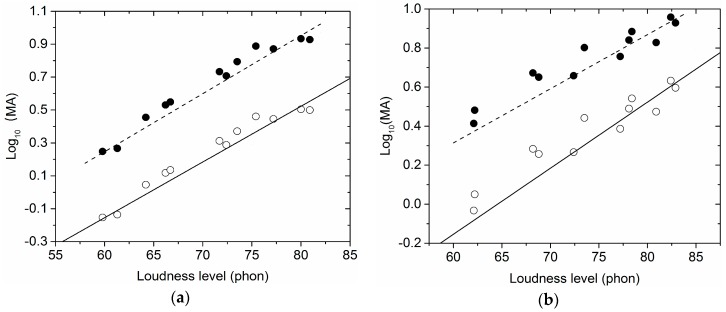
The relationship between L_N_ and logarithmic MA of the 12 noise samples in sample set 1 and sample set 4 before and after calibration. (**a**) sample set 1; (**b**) sample set 4. (●: before calibration; ○: after calibration; solid line: the standard curve; and dotted line: the reference curve).

As shown in Table 3, the *R*^2^ of linear fitting function between L_N_ and logarithmic MA of the 12 noise samples in each sample set (sample sets 1–6) after calibration was consistent with that before calibration (see the shaded part in Table 3). As representatives, Figure 2 gave the relationship between L_N_ and logarithmic MA of the 12 noise samples in sample set 1 and sample set 4. As shown in Figure 2, the relative positions among data points in a sample set were not changed after calibration. It meant that the relative magnitude of MA of noise samples in a sample set would not be changed after calibration using the method proposed in this study.

Annoyance ratings perceived by participants would be influenced by many acoustical factors. It seemed that loudness was the only acoustical factor considered in this calibration method. Was this method useful to calibrate the MA of noise samples whose value of L_N_ was similar and values of some other acoustical factors were different? Actually, in the process of noise exposure, the participant perceived annoyance influenced by all acoustical factors of noise samples and gave a comprehensive evaluation (i.e., MA). Hence, the differences of annoyance ratings of noise samples, whose values of some acoustical factors except L_N_ were different, could also be perceived and identified by participants through listening experiments. According to the analysis results above, the relative magnitude of MA of noise samples in a sample set would not be changed after calibration so the difference of MA caused by the difference of noise samples in acoustical factors except L_N_ would not be eliminated after calibration. Therefore, the method established in this study basing on the relationship between MA and L_N_ was also applicable to calibrating the MA of noise samples whose value of L_N_ was similar and values of some other acoustical factors were different.

### 4.2. The Difference of MA of Identical Noise Sample from Different Sample Sets

**Figure 3:**
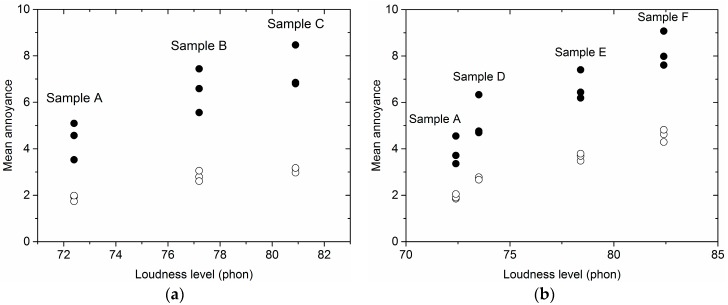
The differences of MA of identical samples (samples A–E) from different sample sets before and after calibration. (**a**) MA of samples A–C obtained from sample sets 1–3; (**b**) MA of samples A, D, E and F obtained from sample set 4–6 (●: before calibration; ○: after calibration).

Figure 3a showed the maximal differences of identical noise samples’ (i.e., samples A–C) MA obtained from sample sets 1–3 decreased to 0.25, 0.45 and 0.20 after calibration from 1.57, 1.88 and 1.66 before calibration, respectively. Furthermore, Table 4 gave the standard deviation and coefficient of variation of identical noise samples’ (i.e., samples A–C) MA obtained from sample sets 1–3. These two values were also markedly decreased after calibration.

Figure 3b showed that the maximal difference of MA for samples A, D, E and F obtained from sets 4–6 decreased to 0.31, 0.21, 0.11 and 0.53 after calibration from 1.21, 1.19, 1.63 and 1.47 before calibration, respectively. Furthermore, Table 5 gave the standard deviation and coefficient of variation of identical noise samples’ (i.e., samples A, D, E and F) MA obtained from sample sets 4–6. These two values were also markedly reduced after calibration. 

**Figure 4:**
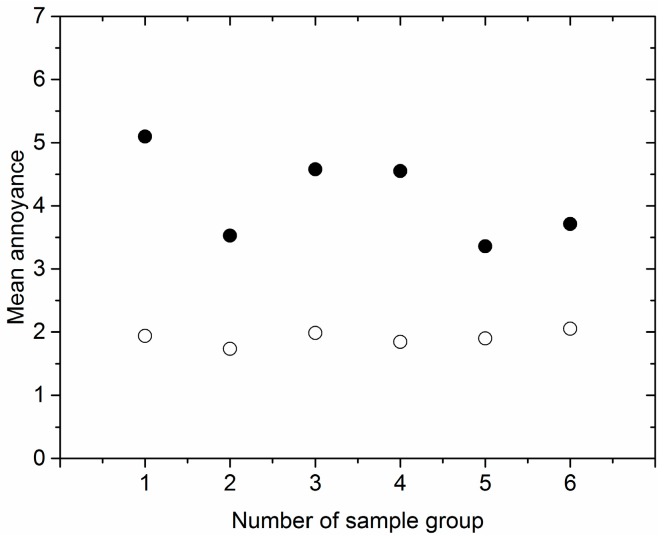
MA of sample A in sample sets 1–6 before and after calibration. (●: before calibration; ○: after calibration).

Sample A existed in all six sample sets. Figure 4 showed that the maximal difference of MA of sample A obtained from sample sets 1–6 decreased to 0.31 after calibration from 1.74 before calibration. Further calculation found that the standard deviation and coefficient of variation of MA of sample A in sample sets 1–6 were 0.638 and 0.101, respectively, before calibration, which decreased to 0.154 and 0.053 after calibration.

It was evident that there were large differences among MA of an identical noise sample from different experimental sample sets before calibration and the differences were markedly decreased after calibration. The results of case study support that the calibration method proposed in this study was applicable to calibrating MA of noise samples from different types of experimental sample sets (both the sets composed of one kind of noise and the sets composed of different kinds of noises). The comparability of MA of the noise samples from different experimental sample sets could be improved after calibration by this method.

### 4.3. The Determination Coefficient of Linear Fitting Functions between PA and MA

As shown in Table 6, the *R*^2^ of linear fitting functions between psychoacoustic annoyance (PA) and MA of the 12 noise samples in each individual set (sample sets 1–6) were 0.830–0.920 before calibration and 0.841–0.929 after calibration, which showed a good linear correlation between PA and MA of noise samples in each sample set.

**Figure 5:**
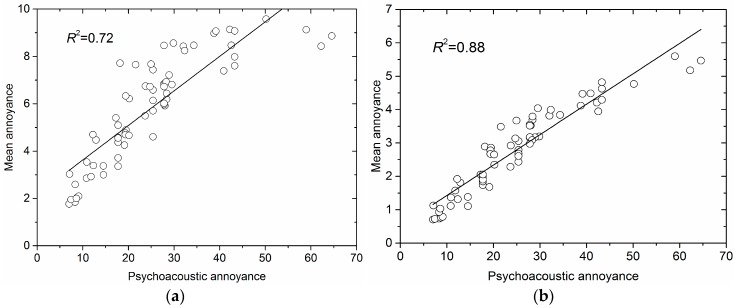
Linear fitting between PA and MA of all noise samples in sample sets 1–6. (**a**) before calibration; (**b**) after calibration.

It could be seen in Table 6 that, compared with those before calibration, the *R*^2^ of linear fitting functions between PA and MA of three mixed sets (mixed sets 1–3, 4–6 and 1–6) after calibration increased to 0.919 from 0.858, 0.878 from 0.770 and 0.881 from 0.722, respectively. As shown in Figure 5, MA of all noise samples in sample sets 1–6 after calibration was more linearly related to PA than that before calibration. The results also showed that the comparability of MA of noise samples from different sample sets was improved after calibration.

## 5. Conclusions

To improve the poor comparability of MA from different studies, this study proposed a calibration method to calibrate the MA of noise samples obtained from listening experiments. Six noise samples sets—half of them were homogenous (transformer noise) and the others were heterogeneous (each set was composed of several kinds of noises)—were selected to examine the applicability of this calibration method. Results show that this method is applicable to calibrating MA of noise samples from different types of noise sample sets. After calibration, the comparability of MA of noise samples from different experimental sample sets can be distinctly improved. 

It should be noted that only several kinds of noise were selected to examine the applicability of this calibration method in this study. However, environmental noises are diverse and there are large differences among acoustical characteristics of different noises. Considering this, the applicability of this method is worth verifying further by more case studies covering all kinds of noise samples with different acoustical characteristics.

## Figures and Tables

**Table 1:** Sources, loudness levels, and energy distribution in different frequency ranges of six identical noise samples (samples A–E).

Sample Number	Source	L_N_/phon	Energy Distribution		
			Low-Frequency Range	Mid-Frequency Range	High-Frequency Range
A	transformer noise	72.4	98.18%	1.50%	0.32%
B	transformer noise	77.2	98.37%	1.35%	0.18%
C	transformer noise	80.9	98.25%	1.70%	0.05%
D	heat pump noise	73.5	34.32%	62.89%	2.79%
E	boiler noise	78.4	98.88%	1.10%	0.02%
F	noise recorded in a workshop	82.4	4.44%	34.37%	61.19%

**Table 2:** The composition of sample sets 1–6.

Number of Sample Set	Range of L_N_/phon	Noise Samples	Reference Sound Samples (7 Pink Noise Samples)	Identical Samples
Sample set 1	59.8–80.9	12 transformer noises	Ranging from 58 phon to 82 phon in 4-phon steps	Samples A–C
Sample set 2	69.7–86.8	12 transformer noises	Ranging from 69 phon to 87 phon in 3-phon steps	
Sample set 3	60.8–88.3	12 transformer noises	Ranging from 58 phon to 88 phon in 5-phon steps	
Sample set 4	62.1–82.9	2 heat pump noises, 2 boiler noises, 3 transformer noises, 5 noises recorded in a workshop	Ranging from 60 phon to 84 phon in 4-phon steps	Samples A, D, E and F
Sample set 5	72.4–91.8	1 boiler noise, 3 heat pump noises, 3 transformer noises, 5 noises recorded in a workshop	Ranging from 70 phon to 94 phon in 4-phon steps	
Sample set 6	62.8–93.4	2 heat pump noises, 3 boiler noises, 3 transformer noises, 4 noises recorded in a workshop	Ranging from 60 phon to 96 phon in 6-phon steps	

**Table 3:** Linear fitting results between L_N_ and logarithmic MA in each sample set.

Sample Set Number	Reference Curves	*R*^2^	*R*^2^ of Linear Fitting Functions for the 12 Noise Samples in Each Sample Set	
			Before Calibration	After Calibration
Sample set 1	log_10_(MA) = 0.035L_N_ − 1.868	0.976	0.960	0.960
Sample set 2	log_10_(MA) = 0.032L_N_ − 1.135	0.943	0.928	0.928
Sample set 3	log_10_(MA) = 0.029L_N_ − 1.432	0.942	0.961	0.961
Sample set 4	log_10_(MA) = 0.028L_N_ − 1.348	0.976	0.909	0.909
Sample set 5	log_10_(MA) = 0.026L_N_ − 1.346	0.903	0.875	0.875
Sample set 6	log_10_(MA) = 0.026L_N_ − 1.413	0.910	0.892	0.892
Sample set 7	**Standard curve**		***R*^2^**	
	log_10_(MA) = 0.034L_N_ − 2.185		0.982	

The background color: it was used to make it convenient for readers to find the data in the table which was noted in the manuscript.

**Table 4:** The standard deviation and coefficient of variation of MA for samples A–C obtained from sample sets 1–3 before and after calibration.

Noise Sample		A	B	C
Standard deviation	Before calibration	0.653	0.768	0.774
	After calibration	0.109	0.184	0.091
Coefficient of Variation	Before calibration	0.148	0.118	0.105
	After calibration	0.058	0.065	0.029

**Table 5:** The standard deviation and coefficient of variation of MA for samples A, D, E and F obtained from sample sets 4–6 before and after calibration.

Noise Sample		A	D	E	F
Standard deviation	Before calibration	0.500	0.524	0.756	0.622
	After calibration	0.088	0.129	0.048	0.218
Coefficient of Variation	Before calibration	0.129	0.078	0.144	0.076
	After calibration	0.045	0.035	0.018	0.048

**Table 6:** The *R*^2^ of linear fitting functions between psychoacoustic annoyance (PA) and MA for individual sets and mixed sets before and after calibration.

Sample Set Number	Individual Set						Mixed Set		
	1	2	3	4	5	6	1–3	4–6	1–6
*R*^2^ (before calibration)	0.902	0.920	0.901	0.830	0.869	0.900	0.858	0.770	0.722
*R*^2^ (after calibration)	0.901	0.929	0.910	0.841	0.877	0.910	0.919	0.878	0.881
